# Supplementary figures and images for: Completing Linnaeus’s inventory of the Swedish insect fauna: Only 5,000 species left?
Source: PLoS One. 2020 Mar 4;15(3):e0228561. doi: 10.1371/journal.pone.0228561 (PMC7055846; doi:10.1371/journal.pone.0228561)

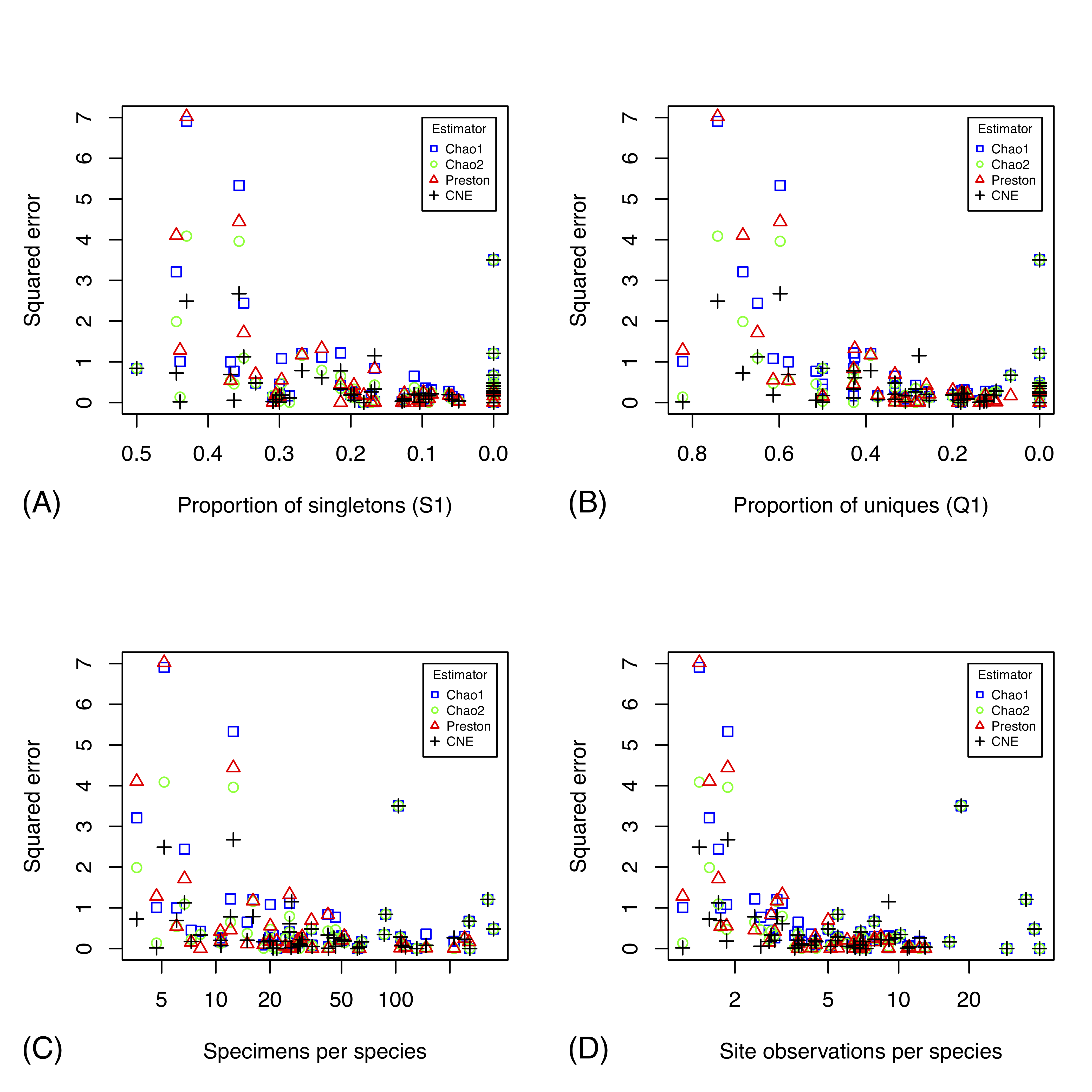

Supplement: S1 Fig — The plots show the accuracy (measured as squared error of log estimates) of the species richness estimates as a function of the proportion of singletons (A), the proportion of uniques (B), the number of specimens per species (C), and the number of site observations per species (D). A “singleton” is a species represented by a single specimen; a “unique” is a species that only occurs at one site. (TIFF) [file pone.0228561.s007.tiff]

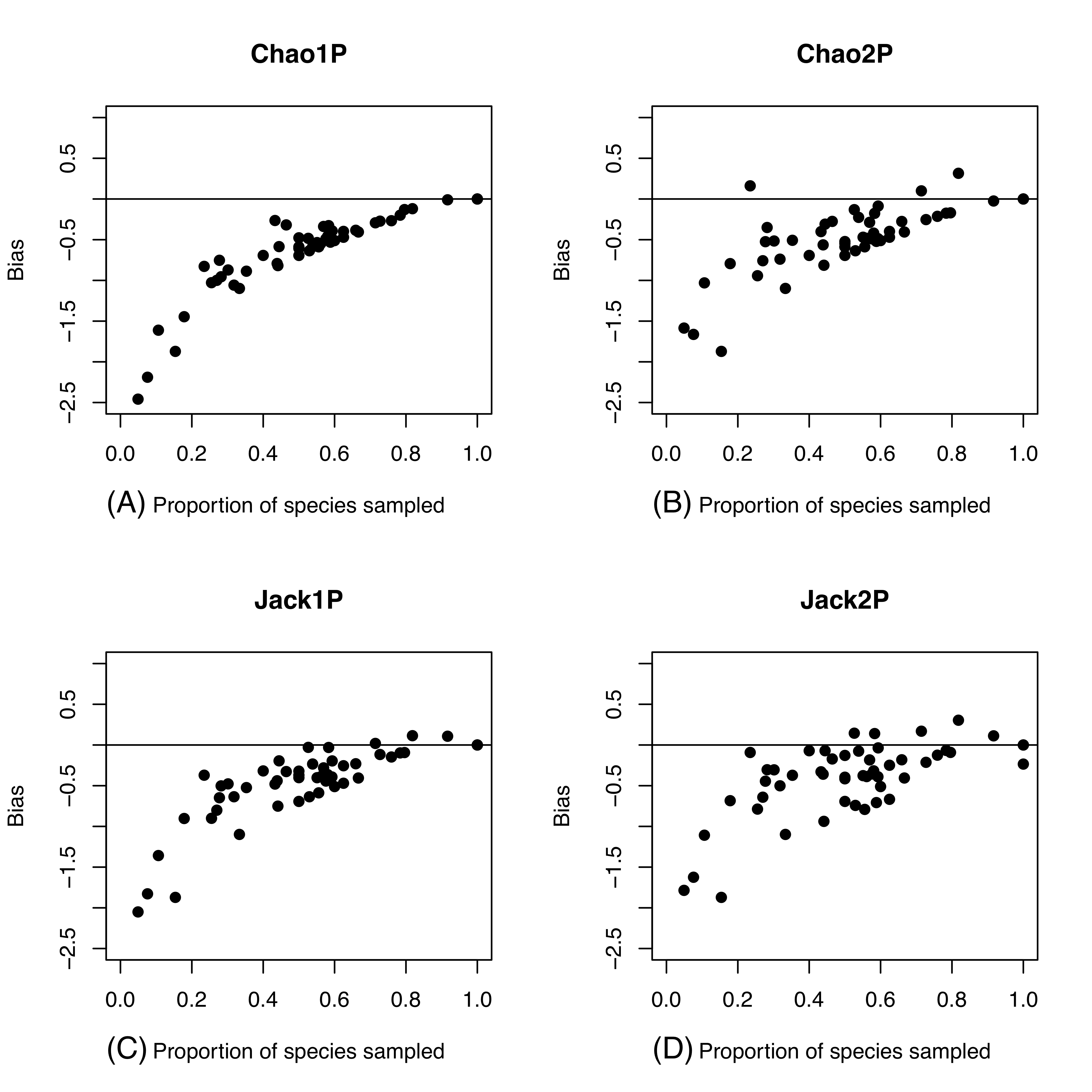

Supplement: S2 Fig — We show the bias in four P-corrected species richness estimators: Chao1P (A), Chao2P (B), Jack1P (C) and Jack2P (D). Bias is measured on the log scale; the horizontal line represents absence of bias. (TIFF) [file pone.0228561.s008.tiff]
