# Supplementary material for: Completing Linnaeus’s inventory of the Swedish insect fauna: Only 5,000 species left?
Source: PLoS One. 2020 Mar 4;15(3):e0228561. doi: 10.1371/journal.pone.0228561 (PMC7055846; doi:10.1371/journal.pone.0228561)
Supplement: Attachment — Submitted filename: Response_to_Reviewers.docx [file pone.0228561.s009.docx]

# Response to Reviewers

Below, we respond to each of the points raised by the editor and the reviewers. Editor and reviewer comments are in italics.

## **Editor**

***Major concerns****: The review process to manuscript PONE-D-19-26826 have been completed. Two reviewers provided interesting comments and helpful suggestions. The first reviser suggets that the section about the development of a new non-parametric estimator (CNE) should go in a separate paper. Although I agree about the need to highlight and discuss this section more clearly, I think this can be done in this paper. Both reviewers also agree that the manuscript structure is unbablanced, with a too short Discussion section in which only part of the results have been discussed. I think, for instance, that the Results section includes a lot of text which should be moved to the Discussion section.*

**Response**: We agree with the editor that it is reasonable to present the CNE estimator in this paper, instead of doing it in a separate paper. It is a simple idea, it was motivated by the present study, and the data in the study serve well to provide an initial test of its performance. In the revised version of the manuscript, we have highlighted the CNE contribution in the abstract, and we provide a more extensive discussion of the CNE estimator in the main text.

We have restructured the paper according to the suggestions of the editor and the reviewers, and we agree that this represents a substantial improvement over the previous version. Specifically, we have changed the order of the sections, such that the “Materials and methods” section now comes immediately after the “Introduction”, as is standard practice in the field. The “Abstract” and “Introduction” sections have been slightly expanded to reflect all parts of the paper. We have removed the methods descriptions in the “Results” section, to avoid repetition, and all discussion of results has been moved to the “Discussion” section. We have also extended the “Results” section, so that all figures and tables are treated in a balanced way, as suggested by Reviewer 2. Among other things, we have shortened some of the figure captions by moving the more descriptive sections to the main text in the “Results” section. Finally, we have expanded the “Discussion” section considerably, to balance the “Results” section. The new “Discussion” section includes a more extensive treatment the topic of metabarcoding, as requested by Reviewer 2.

## **Reviewer #1**

## ***Major concerns:***

*My main concern is that the authors tried to say too much in a single paper, including parts, such as the development of new estimators, that could go to their own paper. And many parts are confusing, probably due to trying to do too much in a single go. The extensive amount of figures and tables reflects this. I could see many ways in which the paper could be shortened and streamlined.*

*As I read the methods before the results (as makes most sense to me, as results without methods are mostly impossible to interpret) the first part of the results sounded a repetition of methods. I advise to swap the order and avoid having two methods sections.*

*The entire section of the development of the new estimator could be on its own paper. Developing a new estimator based on a limited number of tests might be a step too far. Also, the message might be lost as it is not the main goal of the paper. At some point it gets tiring to read so much information and details on so many different methods. Twice…*

With respect to the new CNE estimator, we agree with the editor that it is reasonable to present it in the current paper. It is a simple idea, which can be described succinctly, and the current study provides a good initial test of its performance against other popular estimators. However, we agree with the editor that this contribution should be highlighted better. We now mention it in the abstract, and we provide a more extensive discussion in the main text.

We agree with the reviewer that it was unfortunate to place the “Material and methods” section at the end of the paper. This necessitated some brief repetition of the methods in the “Results” section. In the revised version of the manuscript, we have moved the “Material and methods” section so that it now appears before the “Results” section, and we have removed the repetition of method descriptions there. We have also provided a more balanced treatment of all figures in the revised manuscripts, which will hopefully make it easier to appreciate how these results are connected to the main theme of the paper. We have also introduced subheadings in the “Results” section to better organize the content, hopefully making the paper less confusing and easier to follow.

## ***Minor comments:***

*Ln 163 – In the abstract the authors mention 301 species new to science, not 609. Somehow it seems these are different datasets, but it is not clear.*

In the abstract, we refer to new species that have been described and reported from Sweden in the scientific literature, while the text referred to above gives the number of species that have been putatively identified as new to science in the Malaise trap inventory. We have now clarified this in the text. In the process we also discovered some minor errors in the numbers, which are now corrected. The new passage reads:

“The identified specimens from the Malaise trap inventory (Fig. 1) fall into 4,026 species, of which 689 have been putatively identified as new to science. Of the latter, only 87 have been described so far, forming part of the 301 new species to science recorded from Sweden since the start of the Swedish Taxonomy Initiative (Table S1)*.*”

*Ln 180 – If these are for all groups for which new species were found, how can they represent 29% of the non-recorded fauna and not 100%? Confusing.*

Here, we refer to the fauna assumed by experts to be present in the country but not recorded in 2003. We have modified the sentence to clarify this. The new version of the sentence reads:

“Together, these groups account for 29 % (2,525 of 8,655 species) of the fauna that experts assume is present in the country but that had not been recorded in 2003 (Table 1).”

*Ln 429 – What is the difference between S. borealis and the next five? I recommend to reduce the length of this part.*

There is a distinct difference, in that the family affiliation of *Sciarosoma borealis* is indicated as uncertain, whereas the family-level classification is simply lacking for the other five species. This has been clarified in the revised text, which now reads:

“The family placement of *Sciarosoma* *borealis* (Diptera) is given as uncertain in Dyntaxa; it was included by us in the Diadocidiidae, following the classification in Fauna Europaea [20].”

*Ln 465 – What latitudinal Analysis? And it is Selvagens, not Selagen.*

We have corrected Selagen to Selvagens and apologize for this misspelling in the submitted version of the manuscript. We have now clarified that “latitudinal analysis” refers to an analysis of the latitudinal gradient in the diversity of European insect faunas. The beginning of this paragraph now reads:

“The data were used to analyze the latitudinal gradient in the species richness of kown European insect faunas. In this analysis, we excluded all areas outside of Europe, as well as European areas of countries that mostly lie outside of Europe (Russia and Turkey).”

*Ln 598 – There is a class of estimators that are often found to be less biased than the ones used, the P-corrected versions of Chao1, Chao2, Jack1 and Jack2. These are available in the R package BAT and could be added.*

We thank the reviewer for pointing us to this body of work, which we were not aware of. The P-corrected versions of Chao1, Chao2, Jack1 and Jack2 are now included in the revised version of the manuscript and discussed in the text (S5 Table, S1 Fig and S2 Fig), and the relevant literature is cited. Interestingly, the estimates of the P-corrected versions of Chao2 and Jack2 come close to those of our CNE estimator. *Ln 692 – I am not sure about what this sentence means. The pool is known because it is defined a priori? Was it defined a priori because it was known? Looks like circular reasoning. Rewrite.*

We have rewritten this paragraph to clarify. The previous paragraph read:

“To assess the performance of the species richness estimators, we used test data from two sources. First, we used well-studied groups, defined as those groups where the SMTP inventory has not discovered any new species (Table S4). As the ‘true’ diversity for these groups, we used the number of species in the current expert estimate, which was typically very close to if not identical to the number of known species (Table S4). As a second source of test data, we used the subset of species known in 2003 in the more poorly studied groups, that is, those groups where the SMTP inventory did discover new species (Table S4). For these species pools, the ‘true’ diversity is known as the pool is completely defined a priori. We detected no qualitative difference between these two data sources, except that the first set typically included a smaller number of specimens and represented a much smaller fraction of the expected species pool. We only included groups with more than 100 specimens observed from more than 5 sites in the analyses. The accuracy of the estimators was measured using the squared error and bias of the log estimates, that is, ${(\log\hat{S}-logS)}^{2}$and $\log\hat{S}-\log S$, respectively, where $\hat{S}$ is the estimate and *S* the true value (Figs 2–3).”

The new paragraph reads (rewritten part highlighted):

“To assess the performance of the species richness estimators, we used test data from two sources. First, we used well-studied groups, defined as those groups where the inventory has not discovered any new species (S4 Table). As the ‘true’ diversity for these groups, we used the number of species in the current expert estimate, which was typically very close to if not identical to the number of known species (S4 Table). As a second source of test data, we used the subset of species known in 2003 in the more poorly studied groups, that is, those groups where the inventory did discover new species (S4 Table). **Specifically, the abundance and incidence data for the known species in those groups were used to estimate the total number of known species in the same groups. We detected no qualitative difference between these two data sources (well-known groups versus known species in poorly studied groups)**, except that the first set typically included a smaller number of specimens, and the sample represented a much smaller fraction of the total species pool. We only included groups with more than 100 specimens observed from more than 5 sites in the analyses. The accuracy of the estimators was measured using the squared error and bias of the log estimates, that is, ${(\log\hat{S}-logS)}^{2}$and $\log\hat{S}-\log S$, respectively, where $\hat{S}$ is the estimate and *S* the true value.”

*Ln 694 – Which two data sources?*

We rewrote this part of the paragraph to clarify what the two data sources are. See previous comment.

*Fig 1 – What are trap-days per taxon? In principle all taxa could fall into all traps. Rename or delete.*

We agree that “Trap-days per taxon” may be difficult to understand. We refer to the number of trap days represented by the catches identified to species of the taxon, which is a measure of sampling intensity. We now use “Effort per region” in the figure, and explain this in more detail in the caption. Specifically, we added this sentence to the caption:

“Sampling effort is given as the number of trap days represented by the processed samples for each taxon and region.”

## **Reviewer #2**

## ***Major concerns:***

*The result section is very long presenting data and figures about many question and is not balanced compared to the discussion. The discussion is very short focusing on methodological issues and DNA barcoding and the gap of knowledge and the importance of Hymenoptera and Diptera in comparison to tropical regions and the dominance of Coleoptera. I think the discussion should be extended.*

We agree with the reviewer and have restructured the paper by restructuring the “Results” section and extending the “Discussion” section to cover all topics raised in the paper.

*Given the information and statement in the manuscript that “15 years of focused research on the most poorly known groups in the Swedish insect fauna! And that only “data from the first 165,000 specimens that have been identified, representing about 1 % of the catch” it’s rather depressing that only 1% of the material have been identified during 15 years of work. This naturally comes to the question why no metabarcoding has been done to justify the use of traditional taxonomical identification methods that the authors argue is required. Today metabarcoding is a cheap and well established method to explore species richness in samples that contain thousands or millions of specimens. Lacking such an approach make the study a bit old fashioned and speculative. In the recent literature other studies have come up with contradictory conclusions (among several papers in this topic Morinière et al 2016).*

New methods are developed so quickly that it is easy to lose the time perspective. When the Swedish Malaise trap inventory was started, metabarcoding methods were not available; Sanger-based barcoding of single specimens was still in its early days. If metabarcoding methods had been available, it is likely that they would have been used.

It is true that the number of identified specimens (165,000) on which our study is based is small in comparison to the total catch (20 M specimens), but it is still a sizeable sample. For instance, it more than 1.5 times the number of specimens identified to morphospecies in the Science study of a forest area in Panama cited by us (Bassett et al, 2012). Because our sample targets poorly known groups of Diptera and Hymenoptera, and focuses on a much smaller geographic area, we argue that it is likely to contain more information about the true fauna than the 1 million specimens barcoded in a recent Canadian study cited by us (Hebert et al. 2016). In the revised version of the paper, we try to emphasize these points better.

We also provide more discussion of the pros and cons of metabarcoding compared to our approach. Even though these methods are promising, and will undoubtedly be central in biodiversity research in the future, they do come with their limitations. For instance, the Morinière et al (2016) study mentioned by the reviewer barcoded one Malaise trap catch consisting of 5,000 specimens. Identifying all 20 million specimens in the Swedish Malaise trap inventory using their approach would be challenging and costly, especially if one had to presort all samples to order-level taxonomic fractions as recommended by them. Even processing the 165,000 specimens identified to species from the Swedish Malaise trap inventory using such metabarcoding protocols would require significant resources, if not as much time and resources as traditional taxonomic identification of the material. Nevertheless, we agree with the reviewer that metabarcoding and related genetic methods are the future in large-scale biodiversity studies, and we have indicated this in the revised version of the “Discussion”.

It is difficult for us to assess the claim that the Morinière et al. (2016) paper reaches conclusions that contradict ours. We first thought that this refers to the fact that the Morinière et al (2016) paper fails to detect new species. However, there is virtually no discussion in the paper of the extent to which the detected mOTUs might represent new species to this region of Germany, or to science. They match their COI reads to a comprehensive reference database of Central European insects, but they only specify the number of matches they get, not the number of mOTU clusters that could represent putative new species and that did not match existing BINs. They also say that the German barcoding initiatives GBOL and BFB have built a reference library comprising more than 16,000 animal species, with focal insect groups being Coleoptera, bees, Neuroptera, Heteroptera and Lepidoptera. We note that the focal groups do not include any of the poorly known Diptera or Hymenoptera, which represent the vast majority of all new species discovered in Sweden. Thus, unless we have misunderstood something, there does not appear to be any conflict between our results. Morinièr et al (2016) also mention that they noted a fair amount of diversity of small insects, which was not reflected among the matching BINs:

“Therefore, although we had visual confirmation of a big diversity of small specimens (e.g. Microhymenoptera), this diversity was not detected in either of the samples (presorted or combined)”

This seems to suggest that their results could well be consistent with ours rather than conflicting. In the revised version of the manuscript, we briefly refer to their observation, which we think might be important given that future metabarcoding studies have to be designed so that they do not miss this important fraction of insect diversity.

*In the material and methods it’s assumed that the fauna is constant over time? How about the current extinction rates that are expected to exceed 1000 times higher than in previous era and the colonisation rates in Sweden that should be 1-5% per year depending on taxonomic group? These topics should be mentioned in the manuscript and if it’s not an issue it should be justified. How many species have been extinct from Sweden already? Is only a fraction of the species occurring there still present? Especially this is important for the “true fauna” in Figure 6. Is there a true balanced fauna in Sweden neutral to extinction/colonisation dynamics? Faunas are in most areas in a continuous change especially in recent years?*

These are important points. However, the Swedish Malaise trap data only span over a three-year period, so we argue in the revised manuscript (the initial paragraph of the “Discussion” section) that these factors can be ignored. This paragraph now reads:

“Estimating species richness using statistical methods is notoriously difficult. One problem is that species pools are dynamic over time. Species come and go, and there may also be increases or decreases in the total size of the fauna. All the estimators we explored assume that the species pool is constant during the sampling period. Even though the sampling period of the Malaise trap inventory spanned over three years, we consider this assumption to be justified.”

*Fig. 9-11 is poorly or not at all discussed in the discussion. They deserve a paragraph per figure.*

We agree with the reviewer. In the revised version of the manuscript, we have extended the “Discussion” section to cover the results presented in these figures. We have also described these results in more detail in the “Results” section.

*Why are the recent developments in metabarcoding using the NGS platforms ignored in the manuscript? This deserves more space in the manuscript especially since the discussion is focused on DNA. Among many recent studies in the topic:*

*Reference: Species Identification in Malaise Trap Samples by DNA Barcoding Based on NGS Technologies and a Scoring Matrix. Jérôme Morinière ,Bruno Cancian de Araujo,Athena Wai Lam,Axel Hausmann,Michael Balke,Stefan Schmidt,Lars Hendrich,Dieter Doczkal,Berthold Fartmann,Samuel Arvidsson,Gerhard Haszprunar.* [*https://doi.org/10.1371/journal.pone.0155497*](https://doi.org/10.1371/journal.pone.0155497)

The Discussion on metabarcoding methods has now been extended and the Morinière et al study cited. See above for details.

## ***Minor comments:***

*Macroscopic diversity – what is this? It is commonly used in other disciplines of science. If the authors refer to “referring to objects that are large enough to be seen easily with the naked eye” I doubt it’s this is the case for many animals this paper cover?*

We use macroscopic diversity to refer to the diversity of beings that can be seen with the naked eye, easily or not, which is largely equivalent to the diversity of multicellular organisms. To avoid any misunderstandings, we have now replaced “macroscopic diversity” with “diversity of multicellular life” or “diversity of multicellular organisms” except in one place in the introduction, where it is explained: “the macroscopic floras and faunas (the multicellular organisms)”.

*Row 109-111. Country? I don’t understand? It must be easy to calculate the proportion of known species in Sweden of well know groups (birds/plants/Lepidoptera/Bees etc) compared to the species occurring in Europe. It should be about 25% on average if that’s what the sentence is trying to explain. Half is probably an overestimation and require references (if true). Sweden is situated on young terrain with low average temperatures and have an expected very low-low diversity. Only a few cold adapted groups such as Bombus spp are expected to divide from this pattern.*

We refer to the fact that the Swedish insect fauna comprises 50% of the diversity of multicellular species in Sweden, not that it comprises 50% of the insect diversity of Europe. We have rephrased this sentence slightly to avoid misunderstanding. It now reads:

“Specifically, we focus on Sweden, one of the largest countries in Europe with respect to area, and its insect fauna. The Swedish insect fauna is believed to comprise roughly half the diversity of multicellular organisms in the country.”

*Row141: Collecting?*

We replaced “collecting” with “assembling”, which might be more appropriate.

*Row167: Biogeographic breakdown?*

This appears to be a fairly common expression in English (Merriam-Webster gives one meaning of breakdown as “division into categories”), but it may nevertheless be advisable to avoid using it. Therefore, we have rephrased the first line of the figure caption for Figure 1. It previously read:

“Biogeographic breakdown of the results of the Malaise trap inventory for ten poorly known groups of Hymenoptera and Diptera”

It now reads:

“Results of the Malaise trap inventory by biogeographic region for ten poorly known groups of Hymenoptera and Diptera”

*Figure one is hard to read, increase resolution/font size*

Unfortunately, we submitted a slightly outdated version of Figure 1. We have submitted the correct version now; it includes several minor changes compared to the original version to improve readability. Both Figure 1 and Figure 6 are complex and we would like them to appear well in the final online version of the manuscript. **Please contact the corresponding author if further changes are needed to any of these figures to improve readability**.

*Row 182—185: Reference to the statement “analogous to mark-recapture approximation of
184 population size” Did you use Craigs estimate or Jolly Seber or others? Its hard to find in methods.*

We use the Chapman version of the mark-recapture estimator, as specified in “Material and methods”. The original version of the mark-recapture estimator is known as the Lincoln-Petersen method or the Petersen-Lincoln estimator. We have added this information in the revised version of the paper to make the nature of this estimator clear, in addition to providing the mathematical definition of it, as we did in the previous version of the manuscript. To make it easier to find the definition of the estimators, we now put all the estimator names in the “Material and methods” section in bold.

The Cormack-Jolly-Seber models were developed for population modeling with repeated cycles of mark – release – recapture / resighting, and they do not appear to fit our estimation problem well. The Craig estimator fits abundance data to a Poisson distribution of encounter rates. The estimator does not use information on the number of previously known species, so it is not independent of the estimators based on abundance data or incidence data. If it is applied to the problem of estimating the total species pool, we think it is best thought of as a parametric estimator. However, it is not widely used as a species pool estimator, presumably because it is based on unrealistic assumptions about the species abundance distribution. The parametric Preston estimator, which we use in the paper, should provide more accurate estimates of the species pool than the Craig estimator.

*Row 275: Habitat breakdown ?*

We have rephrased this to avoid the use of “breakdown”. The first sentence of the figure caption now reads:

“Species found in the Malaise trap inventory by habitat”.
